# The effect of short‐term exercise prehabilitation on skeletal muscle protein synthesis and atrophy during bed rest in older men

**DOI:** 10.1002/jcsm.12661

**Published:** 2020-12-21

**Authors:** Benoit Smeuninx, Yasir S. Elhassan, Konstantinos N. Manolopoulos, Elizabeth Sapey, Alison B. Rushton, Sophie J. Edwards, Paul T. Morgan, Andrew Philp, Matthew S. Brook, Nima Gharahdaghi, Kenneth Smith, Philip J. Atherton, Leigh Breen

**Affiliations:** ^1^ School of Sport, Exercise and Rehabilitation Sciences University of Birmingham Birmingham UK; ^2^ Institute of Metabolism and Systems Research University of Birmingham Birmingham UK; ^3^ Centre for Endocrinology, Diabetes and Metabolism Birmingham Health Partners Birmingham UK; ^4^ NIHR Clinical Research Facility, University Hospitals Birmingham NHS Foundation Trust and Institute of Inflammation and Ageing University of Birmingham Birmingham UK; ^5^ Garvan Institute of Medical Research Sydney NSW Australia; ^6^ St Vincents Medical School, UNSW Medicine UNSW Sydney Sydney NSW Australia; ^7^ MRC‐ARUK Centre of Excellence for Musculoskeletal Ageing Research, Clinical, Metabolic and Molecular Physiology University of Nottingham Derby UK; ^8^ MRC‐Arthritis Research UK Centre for Musculoskeletal Ageing Research University of Birmingham UK

**Keywords:** Bed rest, Protein synthesis, Sarcopenia, Muscle

## Abstract

**Background:**

Poor recovery from periods of disuse accelerates age‐related muscle loss, predisposing individuals to the development of secondary adverse health outcomes. Exercise prior to disuse (prehabilitation) may prevent muscle deterioration during subsequent unloading. The present study aimed to investigate the effect of short‐term resistance exercise training (RET) prehabilitation on muscle morphology and regulatory mechanisms during 5 days of bed rest in older men.

**Methods:**

Ten healthy older men aged 65–80 years underwent four bouts of high‐volume unilateral leg RET over 7 days prior to 5 days of inpatient bed rest. Physical activity and step‐count were monitored over the course of RET prehabilitation and bed rest, whilst dietary intake was recorded throughout. Prior to and following bed rest, quadriceps cross‐sectional area (CSA), and hormone/lipid profiles were determined. Serial muscle biopsies and dual‐stable isotope tracers were used to determine integrated myofibrillar protein synthesis (iMyoPS) over RET prehabilitation and bed rest phases, and acute postabsorptive and postprandial myofibrillar protein synthesis (aMyoPS) rates at the end of bed rest.

**Results:**

During bed rest, daily step‐count and light and moderate physical activity time decreased, whilst sedentary time increased when compared with habitual levels (*P* < 0.001 for all). Dietary protein and fibre intake during bed rest were lower than habitual values (*P* < 0.01 for both). iMyoPS rates were significantly greater in the exercised leg (EX) compared with the non‐exercised control leg (CTL) over prehabilitation (1.76 ± 0.37%/day vs. 1.36 ± 0.18%/day, respectively; *P* = 0.007). iMyoPS rates decreased similarly in EX and CTL during bed rest (CTL, 1.07 ± 0.22%/day; EX, 1.30 ± 0.38%/day; *P* = 0.037 and 0.002, respectively). Postprandial aMyoPS rates increased above postabsorptive values in EX only (*P* = 0.018), with no difference in delta postprandial aMyoPS stimulation between legs. Quadriceps CSA at 40%, 60%, and 80% of muscle length decreased significantly in EX and CTL over bed rest (0.69%, 3.5%, and 2.8%, respectively; *P* < 0.01 for all), with no differences between legs. No differences in fibre‐type CSA were observed between legs or with bed rest. Plasma insulin and serum lipids did not change with bed rest.

**Conclusions:**

Short‐term resistance exercise prehabilitation augmented iMyoPS rates in older men but did not offset the relative decline in iMyoPS and muscle mass during bed rest.

## Introduction

Sarcopenia is a condition characterized by skeletal muscle mass and strength loss with increased risk of frailty, falls, metabolic disease, and all‐cause mortality in older individuals.^1^, ^2^ It is estimated that as many as 32 million older individuals across Europe could be diagnosed with sarcopenia by 2045.^3^ Currently, the annual cost to the National Health Service (NHS) of treating age‐related muscle weakness is ~£2.5 billion (~2–3% of budget),^4^ with health care costs ~2–3 times greater in those with muscle weakness.^5^ Thus, sarcopenia poses a major current and future predicted socio‐economic threat.

Periods of disuse and inactivity, typical during illness and hospitalization, result in rapid muscle atrophy and impaired postprandial muscle protein synthesis (MPS) stimulation in older individuals.^6^, ^7^, ^8^, ^9^ The impaired ability of older individuals to fully recover from these acute bouts of inactivity is thought to accumulate over time and contribute to the sarcopenic progression.^10^ This so‐called catabolic crisis model is exemplified by the failure of >50% of hospitalized older individuals to regain pre‐admission mobility levels 12 months after discharge.^11^ Alarmingly, low muscle mass/attenuation and poor physical function at discharge are associated with (i) greater risk of readmission, (ii) longer length of stay, (iii) greater reliance on external care after discharge, and (iv) greater mortality.^12^, ^13^, ^14^ Thus, mitigating disuse‐induced muscle atrophy in older individuals could ultimately delay sarcopenia progression and improve quality of life, with important implications for health care expenditure.

Resistance exercise training (RET) stimulates MPS and can attenuate muscle atrophy when implemented during disuse.^15^, ^16^ However, imposed disuse as a result of illness/co‐morbidities or (planned) elective surgery may preclude older individuals from performing RET. Exercise interventions initiated prior to disuse (‘prehabilitation’) capitalize on a better physical and emotional condition of the patient as compared with peri‐disuse or post‐disuse RET and could potentially benefit a number of clinically relevant outcomes.^17^ Although multi‐modal prehabilitation has been shown to offset the decline in function during disuse and enhance recovery during rehabilitation, findings are inconsistent,^18^, ^19^, ^20^, ^21^, ^22^ potentially because RET stimuli are insufficient (dose and/or time frame) to increase strength and muscle mass in older patients prior to disuse.^23^, ^24^ Furthermore, the effects of RET prehabilitation on muscle morphology and regulatory mechanisms have yet to be explored. Whilst longer‐term high‐volume RET prehabilitation may be required to build a muscle mass and strength reserve in older patients, evidence suggests that several bouts of low‐load high‐volume RET, accumulated over a relatively short period, can augment postprandial MPS responsiveness.^16^, ^25^ Given the possibility that short‐term targeted RET prehabilitation could mitigate disuse‐induced muscle anabolic resistance and, therefore, atrophy, it is imperative to investigate such concise, cost‐saving interventions.

The primary aim of this study was to determine the effects of short‐term unilateral leg RET prehabilitation on quadriceps muscle mass during 5 days of bed rest in older individuals. Using dual‐stable isotope tracer and serial muscle biopsy sampling, we determined integrated myofibrillar protein synthesis (iMyoPS) rates over prehabilitation and bed rest phases, acute postabsorptive and postprandial myofibrillar protein synthesis (aMyoPS) rates, and gene/protein expression of targets known to modulate muscle mass. We hypothesized that RET prehabilitation would augment iMyoPS, gene expression, and anabolic signalling during prehabilitation and that this prior stimulus would offset the expected decline in iMyoPS and muscle mass during bed rest, through greater postprandial aMyoPS responsiveness.

## Methods

### Participants

Ten healthy older men (65–80 years) were recruited through local advertisements and deemed eligible for study participation if they had no history of structured RET within 10 years prior to study participation, were deemed healthy and free of sarcopenia diagnosis as assessed by a general health questionnaire, had a score of ≥9 on the Short Physical Performance Battery test, appendicular lean mass of ≥7.25 kg/m^2^, and a body mass index (BMI) < 30 kg/m^2^.^26^ Participants were excluded from study participation if they had a coagulation disorder, myocardial infarction, artery/vein disease, chronic/systemic illness, or (pre‐)diabetes or underwent hormone replacement therapy. Furthermore, participants were excluded if they currently smoked and consumed any anticoagulant medication or medication that might affect muscle metabolism. Participants were asked to refrain from consuming any nutritional supplements that might affect muscle metabolism during the bed‐rest phase. All participants were informed of the study purpose and procedures and provided their written informed consent. Ethical approval was obtained through the West Midlands—Black Country Research Ethics Committee (16/WM/0483) and was registered at clinicaltrails.gov (NCT04422665; RG_16‐100). The study conformed to the standards outlined by the Declaration of Helsinki (seventh edition).

### Experimental design

**Figure 1:**
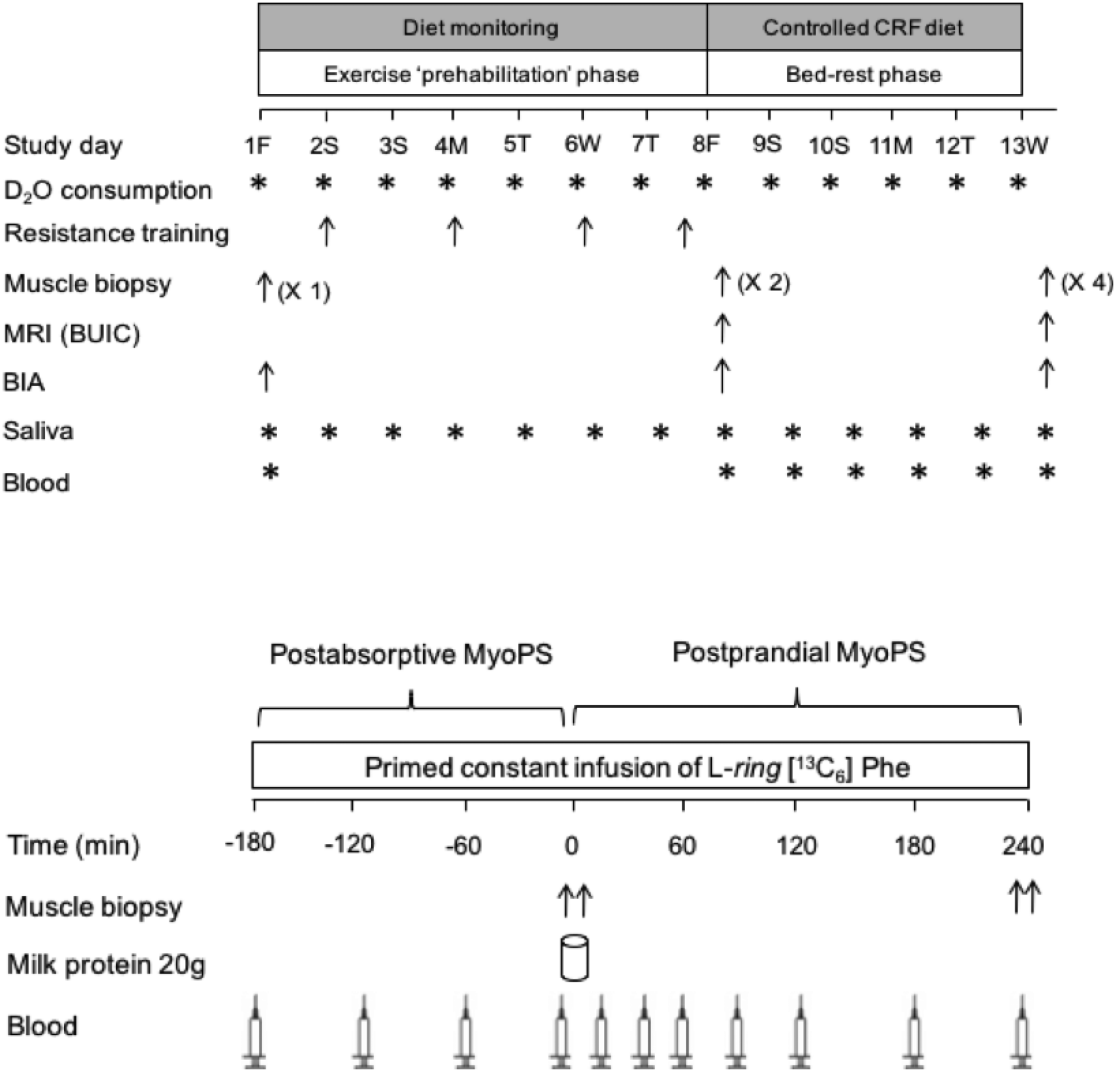
Schematic overview of the longitudinal experimental design (Days 1–13; top) and the acute infusion trial conducted at the end of the bed‐rest period (Day 13; bottom).

After an initial screening visit and obtainment of study consent, participants visited the Wellcome Trust/National Institute for Health Research (NIHR) Clinical Research Facilities (CRF) at the Queen Elizabeth Hospital Birmingham for a preliminary testing visit (Day 1), prehabilitation phase (Days 2–7), mid‐testing visit (Day 8), bed‐rest phase (Days 8–13), and final testing day (Day 13). For each testing visit, participants reported to the CRF at 8 a.m. after an overnight fast (or were already present in the case of Day 13). An overview of the study timeline is depicted in *Figure* 1.

#### Preliminary testing visit (Day 1)

Following an overnight fast, participants provided a single saliva sample after which a baseline muscle biopsy from the vastus lateralis was obtained under 1% lidocaine using the Bergström needle technique, as described in our previous work.^6^, ^27^ The leg to be biopsied first was randomly determined for the first participant and then alternated between subsequent participants. Participant height, body mass, compartmental body composition, estimated one‐repetition maximum (1RM), and physical function were then determined (described subsequently). Participants were then provided with a bolus of deuterated water (D_2_O) and daily top‐up doses (described subsequently) for the measurement of iMyoPS rates. Prior to leaving the CRF, participants were given a 3 day weighed food diary to be completed during the subsequent prehabilitation phase, to determine habitual dietary intake. Participants were fitted with a hip‐worn pedometer and wrist‐worn accelerometer for the remainder of the study to monitor physical activity levels/intensity throughout prehabilitation and bed‐rest phases.

#### Prehabilitation phase (Days 2–7)

Participants reported to the CRF to complete a bout of one‐legged RET on Days 2, 4, 6, and 7. RET was performed by the strongest leg, as determined by the 1RM testing during the preliminary testing visit. Each RET bout consisted of two warm‐up sets at 50% of 1RM, followed by six sets at 75% of 1RM for both the leg extension and leg curl. RET sets consisted of a target 12 repetitions and were separated by 2 min of passive rest. The exercise load was adjusted to maintain a subjective rating of perceived exertion of 8–9 on the modified Borg category‐ratio scale (CR‐10).^28^ RET sessions were conducted at a time convenient for the participant, except for the last RET session, which was performed in the afternoon.

#### Mid‐phase testing visit (Day 8)

On the morning of Day 8 and following an overnight fast, participants reported to the CRF. A skeletal muscle biopsy from the vastus lateralis was obtained from both the exercised (EX) and non‐exercised control leg (CTL). Following this, repeat assessments of height, body mass, and body composition were taken. Finally, participants underwent a magnetic resonance imaging (MRI) scan to determine quadriceps muscle cross‐sectional area (CSA) in both legs (described subsequently).

#### Bed‐rest phase (Days 8–13)

To mimic the effects of a traditional inpatient hospital stay, participants underwent a 5 day period of strict bed rest. Once participants returned from the mid‐testing MRI scan, the bed‐rest period commenced, and participants remained in bed. During the day, participants were allowed to sit up in bed or in a recliner chair. Bathing and sanitary activities were performed in a wheelchair. Accelerometer and pedometer devices were briefly removed during showering. To prevent coagulation disorders and bed sores, participants were given light, non‐weight‐bearing exercises to be performed hourly each day (e.g. knee bends, rolling side to side, and ankle rotations). Participants also wore lower limb compression stockings and received a daily subcutaneous enoxaparin injection (20 mg). To mimic a typical inpatient stay, participants choose from a selection of meals/snacks provided by the CRF during bed rest. Dietary energy and macronutrient intake were not strictly controlled but were closely monitored and logged by CRF nursing staff.

#### Post‐bed‐rest experimental trial (Day 13)

Following an overnight fast, participants were woken up at 6 a.m. A 21 G cannula was inserted in an antecubital vein of both forearms. One cannula was used for serial blood sampling, whilst the other one was used to administer a stable amino acid isotope infusion. After a baseline blood sample was obtained, a primed‐continuous infusion of l‐[ring‐^13^C_6_] phenylalanine was initiated (prime, 2 μmol/kg; infusion, 0.05 μmol/kg, Cambridge Isotope Laboratories, Andover, MA, USA). Blood samples were drawn from the contralateral arm at −185, −120, −60, and −5 min prior to consumption and 20, 30, 60, 90, 120, 180, and 240 min post‐consumption of a milk protein drink (described subsequently). Blood samples were collected in serum separator and ethylenediaminetetraacetic acid (EDTA)‐treated vacutainers (BD Biosciences, Oxford, UK) and centrifuged at 3000 rpm at 4°C, with serum and plasma aliquots stored at −80°C for further analyses. After 150 min of infusion, a muscle biopsy was obtained from the vastus lateralis of both legs. Immediately after biopsy obtainment, participants consumed 18.75 g of milk protein isolate (MyProtein, Cheshire, UK), providing 15 g of milk protein, dissolved in 300 mL of water; 240 min after drink consumption, a second muscle biopsy from the vastus lateralis of each leg was obtained ~3 cm proximal to the first biopsy and indicated the end of the infusion trial. Participants were then fed a meal of their choice and transported in a wheelchair to the MRI scanner for post‐bed‐rest measurement of quadriceps muscle CSA. Obtainment of the MRI scan indicated the end of the bed‐rest phase. Participants walked back to the CRF for a final body composition assessment and consultation/assessment with a trained physiotherapist prior to discharge.

### Experimental procedures

#### Body mass, height, and body composition

Participants' body mass and height were determined in light clothing to the nearest 0.1 kg and 0.1 cm using electronic weighing scales and a stadiometer, respectively. Compartmental body composition was determined using bioelectrical impedance analysis (TANITA BC‐148), with participants holding an electrode in each hand whilst standing barefoot on two other electrodes. Participants were asked to consume 0.5 L of water 30 min before bioelectrical impedance assessments to standardize hydration status, having refrained from fluid consumption beforehand.

#### Maximal strength assessment

Participant knee extensor and flexor estimated 1RM strength was assessed for both legs separately. The leg reaching the highest estimated 1RM was assigned to the exercise intervention. Leg extension 1RM for both right and left leg was determined first, after which the protocol was repeated to determine leg flexor 1RM. Briefly, participants performed a one‐set warm‐up consisting of 12 repetitions at 10 kg. Thereafter, exercise load was gradually increased over subsequent sets until participants were unable to perform >10 repetitions. Increments in exercise load were based on subjective ratings of perceived exertion using the modified Borg category‐ratio scale (CR‐10).^28^ The Brzycki equation was used to estimate 1RM for both knee extensor and flexor strength.

#### D_2_O dosing protocol

The D_2_O dosing protocol consisted of a loading day and 12 maintenance days. On Day 1 of the trial and after providing a background saliva sample, participants consumed a loading dose of 70% D_2_O equalling three times their body mass in millilitres with the aim to label the body water pool to ~0.3 atom per cent excess (APE). The loading dose was split up in 50 mL doses and consumed every 30 min to avoid nausea and light‐headedness. Body water enrichment was maintained in a pseudo steady state using daily top‐ups based upon a 6% per day decay rate. Participants were instructed to provide a daily saliva sample upon waking, followed by consumption of the D_2_O maintenance dose. The D_2_O loading and maintenance protocol was well tolerated, and no adverse effects were reported by the participants.

#### Quadriceps cross‐sectional area

Quadriceps and vastus lateralis CSA was determined on Days 8 and 13 of the experimental trial using a 3 T MRI scanner (Phillips Achieva 3 T scanner) at the Birmingham University Imaging Centre (BUIC). Participants were placed on the scanner bed in a supine position and entered the scanner feet first. To ensure consistent positioning across pre‐bed‐rest and post‐bed‐rest MRI scans and participant comfort, participants' feet were taped to internally rotate the toes, and sandbags were placed over the ankles. To aid alignment of pre‐bed‐rest and post‐bed‐rest MRI scans, cod liver oil tablets were taped every 5 cm on the lateral part of the limb starting at the fibula head running proximal up to the greater trochanter. Accurate placement of cod liver oil capsules was achieved by re‐marking the sites of capsule placement throughout the bed‐rest phase using a non‐toxic pen. Quadriceps and vastus lateralis CSA of both legs was determined from images obtained at 20%, 40%, 60%, and 80% of the length between the top of the patella and the greater trochanter for each participant (OsiriX medical imaging software, OsiriX, Atlanta, USA).

### Sample analyses

#### Plasma amino acids, plasma isotope enrichment, and body water ^2^H enrichment

Plasma [^13^C_6_] phenylalanine enrichment was determined by gas chromatography–mass spectrometry (Model 5973; Hewlett Packard, Palo Alto, CA, USA) by monitoring ion 234/240. Briefly, 100 μL of plasma was diluted 2:1 with acetic acid before being purified through cation‐exchange columns and eluted using 2 M of NH_4_OH. Eluents were dried down under nitrogen and converted to their *N*‐*tert*‐butyldimethyl‐silyl‐*N*‐methyltrifluoracetamide (MTBSTFA) derivative. Leucine and phenylalanine concentrations were measured using internal standards U‐[^13^C_6_] leucine (ions 302/308) and U‐[^13^C_9_‐^15^N] phenylalanine (ions 336/346). Body water enrichment was measured as described by Wilkinson *et al*.^25^ Briefly, 50 μL of saliva was heated in an inverted auto‐sampler vial for 4 h at 100°C and placed upright on ice afterwards to condense extracted body water. This was then transferred to a clean auto‐sampler vial, and a total of 0.1 μL of body water was injected into a high‐temperature elemental analyser (Thermo Finnigan; Thermo Scientific, Hemel Hempstead, UK) connected to an isotope ratio mass spectrometer (Delta V Advantage; Thermo Scientific).

#### Isolation of myofibrillar protein fractions and protein‐bound alanine and ^13^C_6_ phenylalanine enrichment

Myofibrillar proteins were extracted by homogenizing 20–30 mg of muscle in ice‐cold homogenization buffer ﻿[50 mM of Tris·HCl (pH 7.4), 50 mM of NaF, 10 mM of β‐glycerophosphate disodium salt, 1 mM of EDTA, 1 mM of ethylene glycol tetraacetic acid, and 1 mM of activated Na_3_VO_4_ and a complete protease inhibitor cocktail tablet (Roche, West Sussex, UK) at 10 μL/μg tissue and shaken for 10 min. Homogenates were spun at 1000 *g* for 5 min at 4°C, and the supernatant was collected. The myofibrillar fraction of the pellet was solubilized for 30 min at 37°C in 0.3 M of NaOH and separated from the insoluble collagen fraction by centrifugation. Myofibrillar proteins were precipitated using 1 M of perchloric acid and spun down at 3200 *g* for 20 min at 4°C before being hydrolysed overnight in 1 mL of 0.1 M of HCl and 1 mL of Dowex H^+^ Resin. Proteins were eluted from the resin using 2 M of NH_4_OH and dried down at 70°C under a constant nitrogen flow. Amino acids for ^13^C_6_ phenylalanine enrichment were derivatized as their *n*‐acetyl‐*n*‐propyl ester, and labelling was determined using a Thermo Delta V isotope ratio mass spectrometer with a Thermo GC ultra and PAL auto‐sampler, Thermo GC Combustion III interface, and Conflow IV interface. Amino acids for incorporation of deuterium into muscle‐bound alanine were derivatized as their *N*‐methoxycarbonyl methyl esters.^25^ Labelling was determined using chromatography:pyrolysis:isotope ratio mass spectrometry (Delta V Advantage) and ran alongside a standard curve of known dl‐alanine‐2.3.3.3‐*d*_4_ enrichment to ensure measurement accuracy of the machine.

#### Gene expression analysis

Gene expression analysis was performed as previously described.^29^ Briefly, RNA was isolated from ~20 mg of frozen powdered muscle homogenized in 1 mL of TRI Reagent (Sigma Aldrich, Gillingham, UK) and 200 μL of chloroform added to achieve phase separation. The RNA containing supernatant was removed and purified using Reliaprep spin columns (Promega, Madison, Wisconsin, USA). RNA concentration and purity (ratio of the absorbance at 260 and 280 nm and was ≥1.85 for all samples) was determined using a FLUOstar Omega microplate reader; 700 ng of total RNA was reverse transcribed to cDNA in 20 μL volumes using the nanoScript 2 RT kit in combination with oligo (dT) and random primers (Primerdesign, Southampton, UK). cDNA was diluted to 5 ng/μL prior to RT‐qPCR analysis. All analyses were performed in triplicate using Primerdesign custom‐made primer sequences or commercially available 18S, B2M, GAPDH, and ACTB; 5 and 20 ng of cDNA was added, respectively, for housekeeping and human genes of interest to a 20 μL reaction volume. Thermal cycling conditions consisted of 2 min at 95°C, followed by 40 cycles of 10 s at 95°C and 60 s at 60°C. A melt curve was performed (Applied Biosystems, Thermo Fisher, UK) post‐qPCR to assure primer specificity. Results were analysed using Thermo Fisher Connect (Thermo Fisher) and expressed as fold change relative to baseline using the 2^−ΔΔCT^ method. Data were normalized to the geometric mean of the three most stable housekeeping genes (GAPDH, 18S, and ACTB) to minimize variation of the individual housekeeping genes. All gene expression results are presented for *n* = 9 in each group as insufficient tissue was available for one participant. One participant was excluded from the p70S6K gene expression data owing to a value being 15 times the SD. Gene expression targets were measured in muscle biopsy tissue obtained in the postabsorptive state.

#### Protein expression analysis

Protein expression was measured by western blot analysis on the sarcoplasmic protein fraction obtained during myofibrillar protein isolation. Gels were loaded according to the sarcoplasmic protein concentration assessed by the DC protein assay (Bio‐Rad, Hertfordshire, UK), before aliquots of 2 μg/1 μL were prepared in 4× Laemmli sample buffer and ddH_2_O. Samples were boiled for 5 min, and equal amounts of protein (30 μg) were loaded into Criterion™ TGX™ Precast Midi protein gels (Bio‐Rad, Hertfordshire, UK) and separated by sodium dodecyl sulfate–polyacrylamide gel electrophoresis at a constant voltage (100 V for 10 min followed by 150 V for 1 h). Protein samples were transferred to a polyvinylidene difluoride (Whatman, Dassel, Germany) membrane at 100 V for 1 h. The membranes were then incubated overnight (4°C) with appropriate primary antibodies; muscle ring finger protein 1 (MuRF1; sc‐398608, 1:1000 in TBST), muscle atrophy f‐box [MAFbx; AM‐3141, 1:1000 in 5% bovine serum albumin (BSA) TBST] total mechanistic target of rapamycin (mTOR; CST2983, 1:1000 in 3% BSA TBST), phospho‐mTOR^s2448^ (CST2971, 1:1000 in 3% BSA TBST), total ribosomal protein S6 (rpS6; CST2217, 1:1000 in 3% BSA TBST), phospho‐rpS6S^240/244^ (CST5364, 1:500 in 5% BSA TBST), total eukaryotic initiation factor 4E binding protein 1 (4E‐BP1; CST9452, 1:1000 in TBST), phospho‐4E‐BP1^T37/46^(CST9459, 1:500 5% BSA in TBST), total protein kinase B (Akt; CST9272, 1:1000 in TBST), and phospho‐Akt^S473^ (CST4060, 1:1000 5% BSA in TBST). Samples were then incubated for 60 min with a horseradish peroxidase (HRP)‐linked anti‐rabbit (CST7074, 1:10 000 in TBST), anti‐mouse (CST7076, 1:10 000 in 5% BSA TBST), or anti‐rat (CST7077, 1:1000 in 5% BSA TBST) IgG. Following IgG binding, Immobilon western chemiluminescent HRP substrate (Millipore, Watford, UK) was used to quantify protein content, visualized using a:BOX Chemi XT4 imager with GeneSys capture software (Syngene UK, Cambridge, UK). Band quantification was achieved using a Chemi Genius Bioimaging Gel Doc System (Syngene, Cambridge, UK). Values were corrected to a gel control in the first instance before being corrected to the loading control (ponceau). Where appropriate, the phosphorylation of proteins, as a proxy of their activation, was expressed relative to the total amount of each protein.

### Fibre‐type cross‐sectional area

Muscle cross sections (5 μm) were permeabilized in 0.02% Triton X‐100 for 5 min before being incubated for 90 min in 5% normal goat serum. Subsequently, muscle cross sections were incubated overnight in myosin heavy chain type I (IgG2b, BAF8, DSHB, Iowa, USA) and myosin heavy chain type II (IgG1, SC.71, DSHB, Iowa, USA) primary antibodies. The following day, muscle cross sections were washed three times for 5 min in 1× phosphate‐buffered saline (PBS) and incubated in their respective secondary antibodies with wheat germ agglutinin Igg for 90 min. Finally muscle cross sections were washed in 1× PBS, and slides were mounted with Prolong Gold anti‐fade reagent (P36930, Invitrogen). Images were captured using with an Eclipse E600 (Nikon, Badhoevedorp, the Netherlands) and a 20× zoom. All images were analysed using ImageJ Fiji software.

### Plasma insulin, glucose, triglycerides, and non‐esterified fatty acids

Plasma insulin concentrations were analysed using a commercially available enzyme‐linked immunosorbent assay according to the manufacturer's instructions (R&D Systems, MN, USA). Plasma glucose was measured using a Roche Cobas 8000 analyser (Roche Diagnostics, Basel Switzerland). Serum total cholesterol, high‐density lipoprotein cholesterol (HDL‐C), triglyceride (TG), and non‐esterified fatty acid (NEFA) (Randox, London, UK, for all) were analysed using an ILAB 650 Clinical Chemistry Analyser.

### Calculations

The iMyoPS and aMyoPS fractional synthetic rate (FSR) was determined as percentage per hour and percentage per day for [^13^C_6_] phenylalanine and [^2^H] alanine, respectively, with the use of the precursor‐product equation, as previously described.^6^, ^25^ The use of tracer naïve participants allowed us to use the pre‐infusion plasma ^13^C_6_ phenylalanine enrichment as a proxy for basal muscle protein enrichment for measurement of postabsorptive aMyoPS rates. This approach has been validated for use in older individuals.^30^

### Statistical analyses

Anthropometric, physical activity, dietary characteristics, and blood hormone/analytes were analysed using a paired‐samples *t*‐test. Quadriceps CSA, fibre‐type morphology, gene expression, and iMyoPS and aMyoPS were analysed using a two‐way repeated‐measures ANOVA (condition × time) with condition (CTL vs. EX) and time (pre‐bed rest vs. post‐bed rest). Body water ^2^H enrichment, plasma amino acid, and ^13^C_6_ phenylalanine were analysed using a one‐way repeated‐measures ANOVA with time as the within‐subject factor. Delta and percentage change in quadriceps CSA, iMyoPS, and aMyoPS for CTL and EX were analysed using a Student's *t*‐test. Bonferroni post‐hoc tests were performed to correct for multiple comparisons when a significant condition × time interaction was identified. All analyses were performed using SPSS 26 (SPSS, Chicago, IL, USA). Significance was set at *P* ≤ 0.05. All data are presented as mean ± SD unless otherwise indicated.

## Results

### Anthropometric, physical activity, and dietary characteristics

Participant body mass and BMI did not change from pre‐bed rest to post‐bed rest. However, following bed rest, a decrease in relative fat‐free mass (~5%; *P* = 0.02) and appendicular lean mass (~6%; *P* = 0.002) and an increase in relative fat mass (~13%; *P* = 0.02) were observed. Average daily step‐count and percentage of daily time spent performing light‐intensity and moderate‐intensity activities were significantly lower during bed rest compared with habitual levels (*P* < 0.001 for all). Percentage of daily sedentary time increased significantly during bed rest (~27%; *P* < 0.001), whilst light and moderate activity decreased (both *P* < 0.001). Percentage of daily vigorous activity did not significantly change during bed rest. During bed rest, total energy (*P* = 0.025), dietary protein (*P* = 0.01), and fibre (*P* = 0.001) intake significantly decreased from habitual levels. Anthropometric, physical activity, and dietary characteristics are presented in *Table* 1.

**Table 1:** Participant anthropometric, activity, and dietary characteristics before and during/after bed rest

Characteristic	Pre‐bed rest	Peri‐to‐post‐bed rest	*P*‐value
Age (years)	71.5 ± 4.0		
Height (m)	1.77 ± 0.07		
Weight (kg)	79.6 ± 9.0	79.0 ± 8.4	0.212
BMI (kg/m^2^)	25.5 ± 2.8	25.3 ± 2.7	0.207
Body Fat (%)	23.3 ± 4.9	27.1 ± 4.0*	0.021
Fat‐free mass (%)	76.7 ± 5.0	72.9 ± 4.0*	0.022
ALM (kg/m^2^)	7.90 ± 0.96	7.45 ± 0.85	0.002
SPPB	12 ± 0.1		
Daily step‐count	10 177 ± 3695	134 ± 161*	<0.001
Sedentary activity (%)	70.67 ± 9.38	89.40 ± 6.87*	<0.001
Light activity (%)	12.32 ± 3.85	4.83 ± 2.30*	<0.001
Moderate activity (%)	16.47 ± 6.85	5.70 ± 4.98*	<0.001
Vigorous activity (%)	0.67 ± 1.21	0.08 ± 0.11	0.014
Total energy intake (kcal)	2451 ± 750	1990 ± 369*	0.025
Protein intake (g/kg/day)	1.37 ± 0.47	0.97 ± 0.25*	0.004
CHO intake (g/kg/day)	2.71 ± 1.08	2.73 ± 0.54	0.932
Fat intake (g/kg/day)	1.19 ± 0.49	1.12 ± 0.24	0.521
Fibre intake (g/kg/day)	0.39 ± 0.19	0.10 ± 0.06*	<0.001
Alcohol Intake (g/kg/day)	0.09 ± 0.14	0.00 ± 0.00	0.085

Step‐count, physical activity, and dietary intake data are daily averages obtained over 5 days of bed rest. Values are means ± SD for *n* = 10 participants.

ALM, appendicular lean mass; BMI, body mass index; SPPB, Short Physical Performance Battery.

* Significantly different from corresponding pre‐bed‐rest value (*P* < 0.05).

### Maximal strength and resistance training parameters

Estimated 1RM strength was 64.7 ± 14.8 and 58.6 ± 7.7 for the leg extension and leg curl machines, respectively. The average total training volume over the four sessions of prehabilitation RET was 22 407 ± 3340 kg, performed at an average Borg CR‐10 rating of 8.1 ± 1.0. RET prehabilitation data are presented in *Table* 2.

**Table 2:** Leg strength and resistance training parameters for leg extension and leg curl exercises

Parameter	Leg extension	Leg curl
Estimated 1RM (kg)	64.7 ± 14.8	58.6 ± 7.7
Avg load per set (kg)	41.3 ± 8.7	36.7 ± 4.9
Total load (kg)	986 ± 203	880 ± 118
Avg repetitions per set	12.0 ± 0.1	12.0 ± 0.0
Total repetitions	287 ± 1	288 ± 1
Avg volume per set (kg)	494 ± 106	440 ± 58
Total volume (kg)	11 857 ± 2541	10 550 ± 1390
T‐U‐T per set (s)	23.0 ± 3.5	23.0 ± 2.9
T‐U‐T total (s)	551.8 ± 83.8	551.0 ± 70.5
Avg Borg CR‐10	8.2 ± 1.0	8.0 ± 0.9

Values are means ± SD for *n* = 10 participants.

1RM, one‐repetition maximum strength; T‐U‐T, time under tension.

### Quadriceps cross‐sectional area and fibre‐type morphology

**Figure 2:**
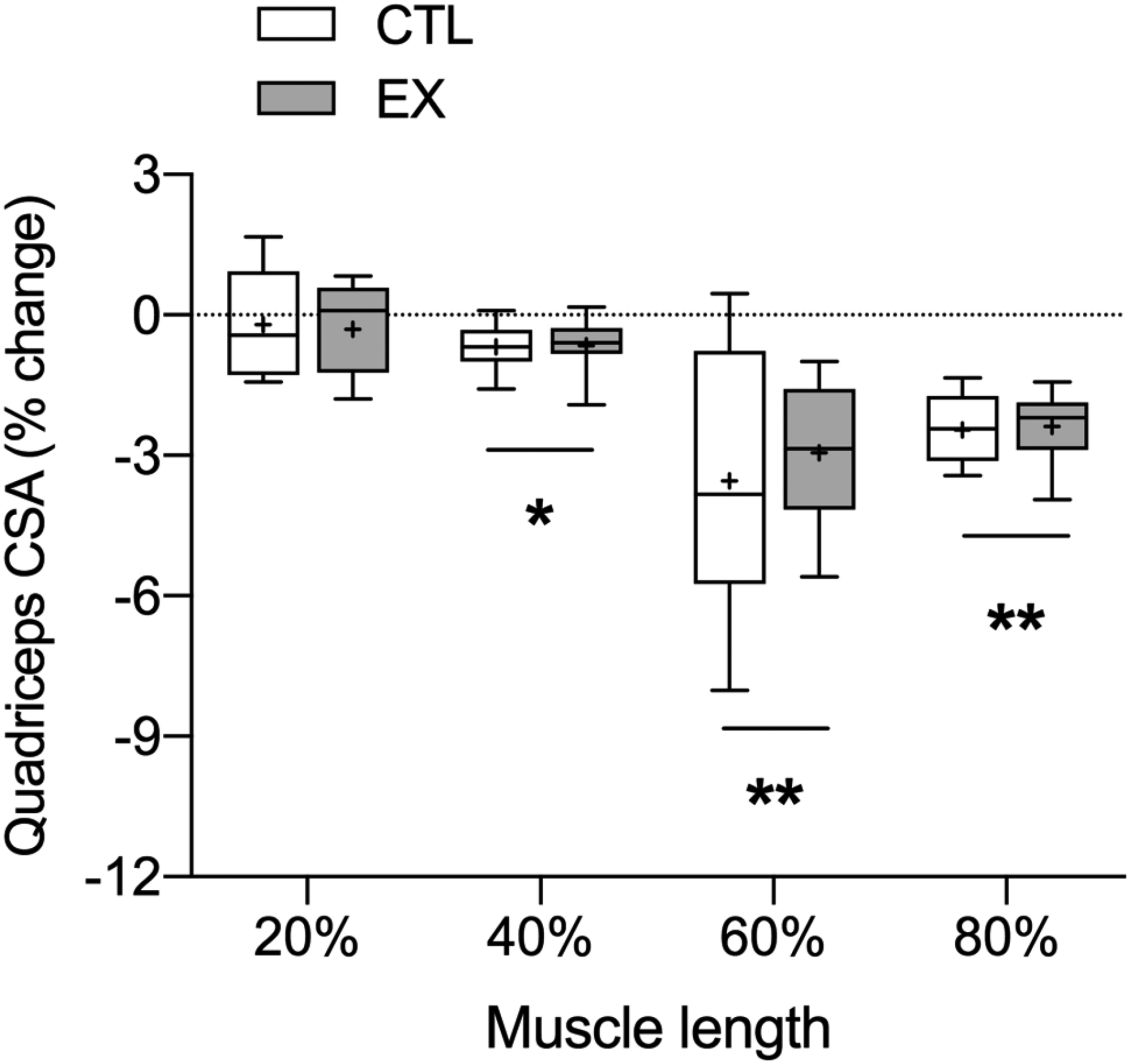
Percentage change in quadriceps cross‐sectional area during 5 days of bed rest in healthy older men in a leg that had undergone resistance exercise prehabilitation over the preceding 7 days (EX) or the contralateral non‐exercised control leg (CTL). Magnetic resonance imaging was obtained at 20%, 40%, 60%, and 80% of the length between the top of the patella and the greater trochanter (distal to proximal). Between‐leg differences were analysed using a Student's paired *t*‐test. Boxes represent the 25th to 75th percentiles, error bars represent SEM, and horizontal lines and crosses within boxes represent median and mean values, respectively (*n* = 9). Significance was set at *P* < 0.05. A significant reduction in quadriceps cross‐sectional area (CSA) was noted at 40%, 60%, and 80% of muscle length (*P < 0.01, ** *P* < 0.001), with no between‐group differences observed at any length.

Quadriceps CSA significantly decreased from pre‐to‐post bed rest at 40%, 60%, and 80% (*P* < 0.01 for all), but not at 20% of the distance measured between the top of the patella and greater trochanter in CTL and EX. Vastus lateralis CSA significantly decreased from pre‐to‐post bed rest at 40% and 60% (*P* < 0.05 for all), but not at 20% and 80% of the distance measured between the top of the patella and greater trochanter in CTL and EX. No differences for quadriceps or vastus lateralis CSA between EX and CTL were found before or after bed rest. Furthermore, there was no significant difference between EX and CTL in the relative change in quadriceps CSA or vastus lateralis CSA from pre‐to‐post bed rest at any anatomical muscle length. Quadriceps and vastus lateralis CSA are presented in *Table* 3 and *Figure* 2. To determine fibre‐type CSA, a total of 77 ± 19 fibres were analysed for each biopsy. No significant group, time, or interaction effects were found for fibre‐type CSA (*Table* 3, representative images in *Figure* S1).

**Table 3:** Quadriceps and fibre cross‐sectional area for non‐exercised control and exercised legs measured prior to and following 5 days of bed rest

	CTL			EX		
	Pre‐bed rest	Post‐bed rest	*P*‐value	Pre‐bed rest	Post‐bed rest	*P*‐value
Quadriceps CSA 20% (mm^2^)	4770 ± 649	4760 ± 641	0.542	4787 ± 600	4774 ± 620	0.465
Quadriceps CSA 40% (mm^2^)	6823 ± 677	6776 ± 663*	0.004	6855 ± 692	6809 ± 671*	0.005
Quadriceps CSA 60% (mm^2^)	7168 ± 826	6917 ± 717*	0.001	7260 ± 868	7040 ± 783*	0.002
Quadriceps CSA 80% (mm^2^)	5086 ± 759	4963 ± 733*	<0.001	5148 ± 679	5027 ± 679*	<0.001
VL CSA 20% (mm^2^)	1198 ± 145	1186 ± 119	0.429	1203 ± 116	1199 ± 138	0.560
VL CSA 40% (mm^2^)	1889 ± 193	1860 ± 157*	0.021	1919 ± 184	1892 ± 193*	0.018
VL CSA 60% (mm^2^)	2246 ± 338	2158 ± 325*	<0.001	2316 ± 300	2221 ± 357*	0.007
VL CSA 80% (mm^2^)	1290 ± 266	1271 ± 185	0.083	1338 ± 202	1315 ± 174	0.064
Type I fibre CSA (μm^2^)	6162 ± 2100	5638 ± 1175	0.397	6222 ± 1689	5703 ± 2281	0.466
Type II fibre CSA (μm^2^)	6089 ± 2343	5743 ± 1156	0.505	5863 ± 1689	5577 ± 1530	0.633

Values are means ± SD for *n* = 10 participants.

CSA, cross‐sectional area; VL, vastus lateralis.

* Significantly different from corresponding pre‐bed‐rest value (*P* < 0.05).

### Blood hormones and analytes

No significant differences between fasting pre‐bed‐rest and post‐bed‐rest values were found for total cholesterol to HDL ratio and concentrations of plasma insulin, glucose, serum total cholesterol, serum HDL, serum non‐HDL, serum NEFA, and serum TG. Homeostatic Model Assessment of Insulin Resistance (HOMA‐IR), derived from fasting plasma glucose and insulin, did not differ between pre‐bed rest and post‐bed rest. Blood hormone and analyte data are presented in *Table* 4.

**Table 4:** Fasting blood hormone and analyte concentrations prior to and following 5 days of bed rest

	Pre‐bed rest	Post‐bed rest	*P*‐value
Plasma insulin (pmol/L)	38.8 ± 19.4	46.4 ± 22.6	0.094
Plasma glucose (mmol/L)	5.29 ± 0.72	5.45 ± 0.82	0.116
HOMA‐IR	1.54 ± 0.90	1.92 ± 1.08	0.070
Serum total cholesterol (mmol/L)	5.11 ± 1.24	4.50 ± 0.77	0.073
Serum HDL‐C (mmol/L)	1.65 ± 0.44	1.44 ± 0.38	0.588
Non‐HDL‐C (mmol/L)	3.46 ± 1.12	3.06 ± 0.68	0.124
Total cholesterol:HDL‐C ratio	3.20 ± 0.73	3.26 ± 0.78	0.588
Serum NEFA (mmol/L)	0.49 ± 0.14	0.46 ± 0.17	0.390
Serum triglycerides (mmol/L)	1.07 ± 0.36	1.11 ± 0.46	0.602

Values are means ± SD for *n* = 10 participants.

HDL‐C, high‐density lipoprotein cholesterol; HOMA‐IR: Homeostatic Model Assessment of Insulin Resistance; NEFA, non‐esterified fatty acids.

### Plasma amino acid, ^13^C_6_ phenylalanine, and ^2^H body water enrichment

**Figure 3:**
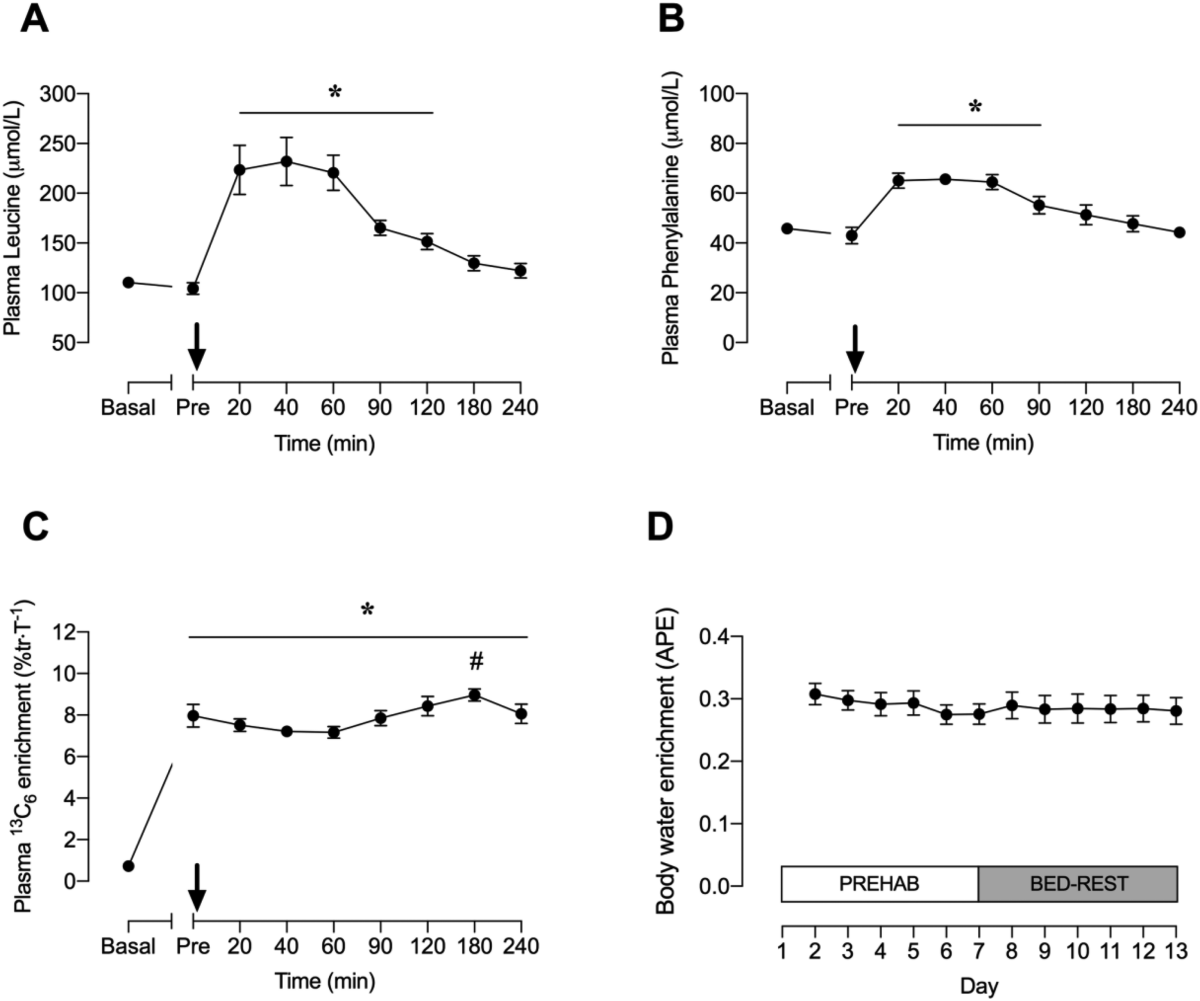
Plasma leucine concentration *(A)*, phenylalanine concentration *(B)*, and plasma ^13^C_6_ phenylalanine enrichment *(C)* measured in the experimental infusion trial (Day 13) and body water ^2^H enrichment measured in daily saliva samples over the course of the study (Days 1–13). *(A–C)* The black arrow indicates the point at which a 15 g bolus of milk protein isolate was consumed. Plasma and saliva data were analysed using a one‐way repeated‐measures ANOVA with time as the within‐subject factor. Values are means ± SEM (*n* = 10 for all panels). Significance was set at *P* < 0.05. Plasma leucine and phenylalanine concentrations were significantly increased above basal‐fasted and pre‐drink values at 20–120 and 20–90 min post‐drink, respectively (*P < 0.05 for both). Plasma ^13^C_6_ phenylalanine enrichment was significantly increased above basal‐fasted values at pre‐drink and remained elevated for the duration of the experiment (*P < 0.05). Plasma ^13^C_6_ phenylalanine enrichment at 180 min post‐drink was significantly >20, 40, 60, and 90 min post‐drink (#*P* < 0.05).

Plasma leucine concentrations were significantly elevated above postabsorptive values at 20, 40, 60, 90, and 120 min post‐drink consumption (*Figure* 3(A)), whilst plasma phenylalanine concentrations were significantly elevated above postabsorptive values at 20, 40, 60, and 90 min post‐drink consumption (*Figure* 3(B)). Plasma ^13^C_6_ phenylalanine enrichment significantly increased above basal values 180 min after initiation of the stable isotope tracer infusion and remained elevated for the duration of the trial (*P* < 0.001; *Figure* 3(C)). Plasma ^13^C_6_ phenylalanine enrichment at 180 min post‐drink consumption was significantly different to values at 20, 40, 60, and 90 min post‐drink consumption. Nonetheless, linear regression analysis revealed that the ^13^C_6_ phenylalanine enrichment slopes in both groups were not significantly different from zero. Body water ^2^H enrichment, assessed via saliva, was 0.31 ± 0.02 APE at 24 h after the first bolus D_2_O dose (*Figure* 3(D)). Body water ^2^H enrichment averaged 0.29 ± 0.02 APE over the duration of the study. Linear regression analysis indicated that the slope of the body water enrichment curve was significantly different from zero (*r* ^2^ = 0.41; *P* = 0.025).

### Integrated myofibrillar protein synthesis

**Figure 4:**
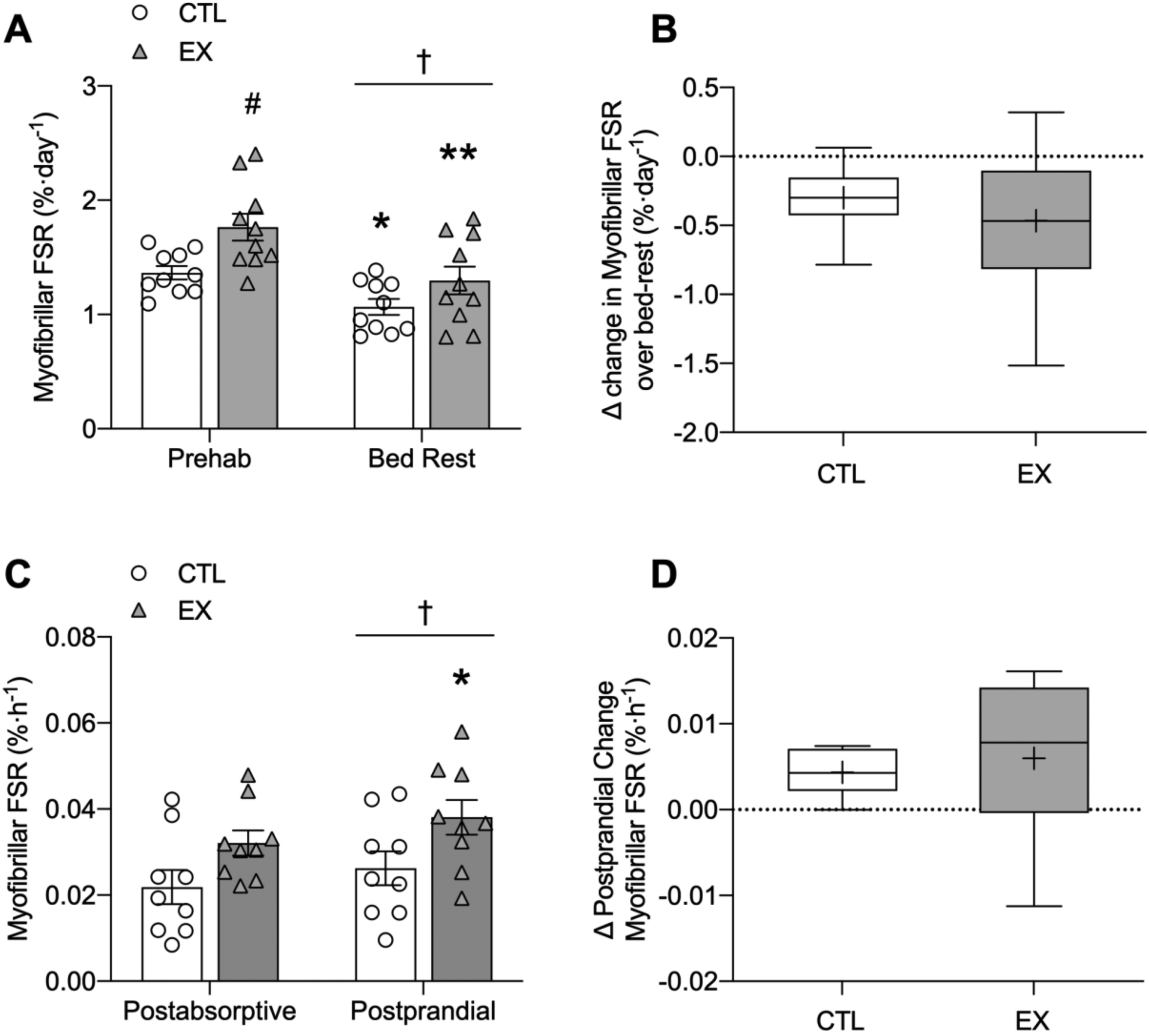
Integrated myofibrillar protein fractional synthesis rates over the course of 7 days of resistance exercise prehabilitation and 5 days of bed rest in exercised (EX) and non‐exercised (CTL) legs in healthy older men *(A)*. Delta change in integrated myofibrillar protein fractional synthesis rates from prehabilitation to bed rest in EX and CTL *(B)*. Acutely measured myofibrillar protein synthesis rates in the postabsorptive and postprandial state, after ingestion of 15 g of milk protein, in EX and CTL *(C)*. Delta change in acute myofibrillar protein fractional synthesis rates from postabsorptive to postprandial state in EX and CTL *(D)*. Values in *(A)* and *(C)* are means ± SEM and individual participant data (*n* = 10). *(B)* and *(D)* The boxes represent the 25th to 75th percentiles, error bars represent SEM, and horizontal lines and crosses within boxes represent median and mean values, respectively (*n* = 10). Data in *(A)* and *(C)* were analysed using a two‐way repeated‐measures ANOVA (condition × time) with condition (CTL vs. EX) and time (prehabilitation vs. post‐bed rest). Data in *(B)* and *(D)* were analysed using a Student's *t*‐test. Bonferroni post‐hoc tests were performed to correct for multiple comparisons when a significant condition × time interaction was identified. Significance was set at *P* < 0.05. Integrated myofibrillar protein synthesis (iMyoPS) was higher in EX vs. CTL during prehabilitation (#*P* < 0.01) and was lower during bed rest vs. prehabilitation in EX (**P < 0.01) and CTL (*P < 0.05), with no difference between groups. Acute postabsorptive and postprandial myofibrillar protein synthesis (aMyoPS) was stimulated above postabsorptive values in EX (*P < 0.05), with no differences between groups. † indicates a significant time effect of bed rest vs. prehabilitation (*P* < 0.05).

A significant main effect for time was found (*P* = 0.001), whilst no interaction effect was observed. Rates of iMyoPS were significantly elevated in EX compared with CTL during the prehabilitation phase (1.36 ± 0.18%/day vs. 1.76 ± 0.37%/day; *P* = 0.007). Both CTL and EX iMyoPS rates significantly decreased during bed rest compared with prehabilitation to 1.07 ± 0.22%/day (*P* = 0.037) and 1.30 ± 0.38%/day (*P* = 0.002), respectively. iMyoPS rates did not differ between EX and CTL over the bed‐rest phase. The decrease in iMyoPS from prehabilitation to bed‐rest values was not significantly different between EX and CTL (*Figure* 4(A) and 4(B)).

### Acute myofibrillar protein synthesis

A significant main effect for time (*P* = 0.005) was found with no apparent interaction effect. Whilst not significant, there was a trend for greater aMyoPS rates in EX vs. CTL in the postabsorptive (0.022 ± 0.012%/h vs. 0.032 ± 0.009%/h; *P* = 0.054) and postprandial (0.026 ± 0.012%/h vs. 0.038 ± 0.012%/h; *P* = 0.052) states. aMyoPS rates significantly increased from the postabsorptive to postprandial state in EX only (*P* = 0.018). The postprandial change in aMyoPS from the postabsorptive state did not differ between EX and CTL (*Figure* 4(C) and 4(D)).

### Gene expression

**Figure 5:**
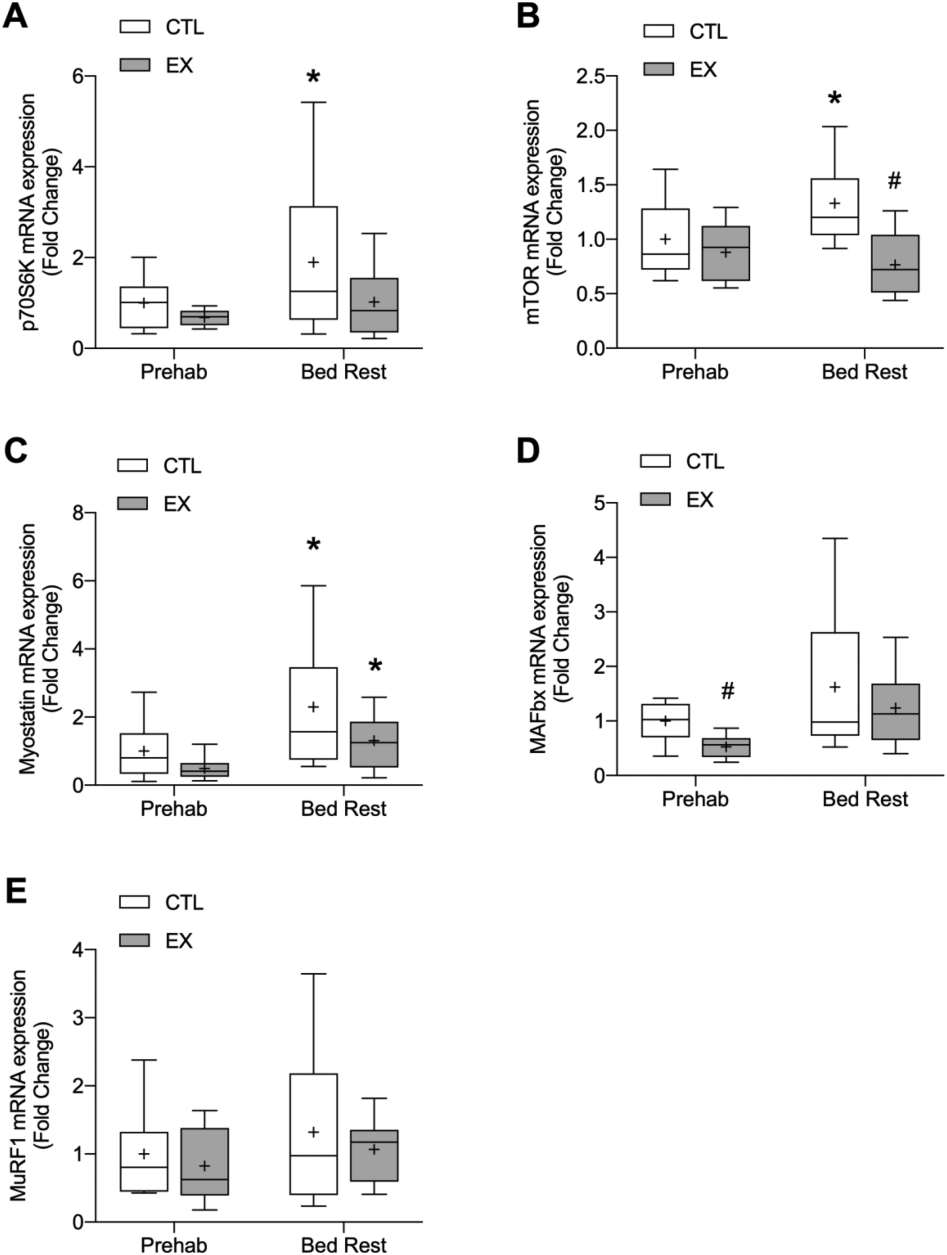
Changes in mRNA expression of p70S6K *(A)*, mTOR *(B)*, myostatin *(C)*, MAFbx *(D)*, and MuRF1 *(E)* after 7 days of resistance exercise prehabilitation and a subsequent 5 days of bed rest in older men. Data are expressed as the fold change from levels measured in non‐exercised control leg (CTL) after prehabilitation, which was normalized to a value of 1. All targets were measured in muscle biopsy tissue obtained in the postabsorptive state. Boxes represent the 25th to 75th percentiles, error bars represent SEM, and horizontal lines and crosses within boxes represent median and mean values, respectively [*n* = 9 for all targets except p70S6K (*n* = 8)]. Significance was set at *P* < 0.05. There was a significant increase in p70S6K and mTOR expression after bed rest compared with prehabilitation for CTL only (*P < 0.05 for both). There expression of mTOR after bed rest was significantly lower in EX vs. CTL (#*P* < 0.05). Myostatin expression was greater after bed rest compared with prehabilitation in EX and CTL (*P < 0.05) with no difference between legs. MAFbx expression was greater in CTL vs. EX after prehabilitation (#*P* < 0.05). There was a significant main effect of time on MAFbx expression from prehabilitation to bed rest (*P* = 0.016).

Both and p70S6K1 (*Figure* 5(A)) and mTOR (*Figure* 5(B)) gene expression was significantly increased after bed rest compared with prehabilitation in CTL only (*P* = 0.026 and *P* = 0.047, respectively). Whilst no differences between EX and CTL were found for p70S6K1, mTOR gene expression was significantly higher in CTL vs. EX after bed rest (*P* = 0.002). Myostatin mRNA expression was significantly increased in both CTL (*P* = 0.001) and EX (*P* = 0.023) following bed rest, with no difference between EX and CTL (*Figure* 5(C)). In regard to genes associated with proteolysis, only MAFbx (*Figure* 5(D)) was significantly higher in CTL compared with EX following prehabilitation (*P* = 0.004). There was a significant main effect of time on MAFbx expression from prehabilitation to bed rest (*P* = 0.016).

No significant group, time, or interaction effects were found for MuRF1 mRNA expression (*Figure* 5(E)).

### Protein expression

**Figure 6:**
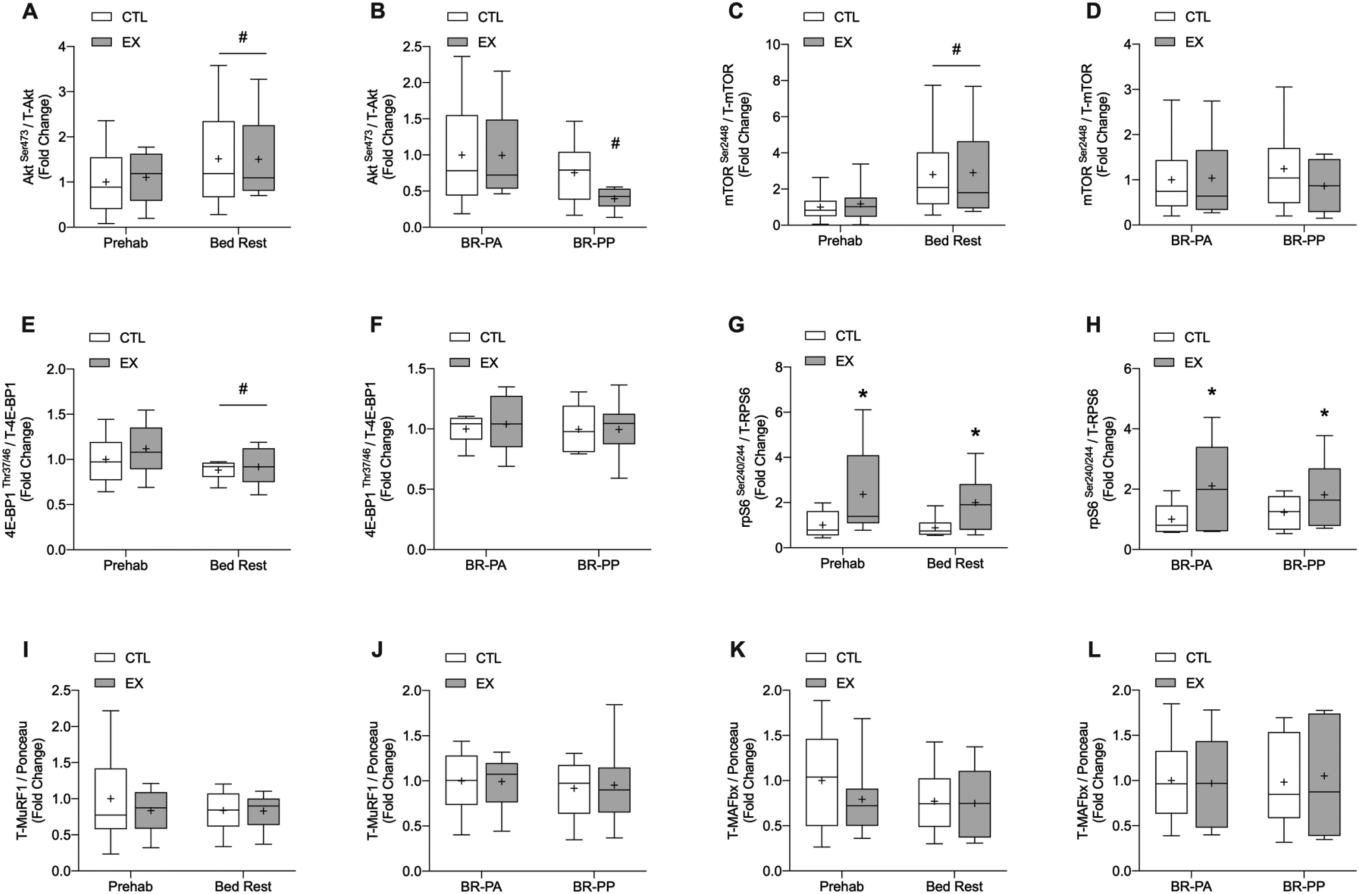
Changes in protein expression of Akt^S473^, mTOR^S2448^, 4E‐BP1^T37/46^ and rpS6^S240/244^, MuRF1, and MAFbx following 7 days of resistance exercise prehabilitation (EX) and a subsequent 5 days of bed rest in older men. Akt^S473^, mTOR^S2448^, 4E‐BP1^T37/46^, and rpS6^S240/244^ are expressed relative to respective total protein expression. MuRF1 and MAFbx are expressed relative to ponceau loading control. *(A)*, *(C)*, *(E)*, *(G)*, *(I)*, and *(K)* show protein expression in EX and CTL after exercise prehabilitation and after bed rest and are expressed as the fold change from levels measured in control (CTL) after prehabilitation, which was normalized to a value of 1 (all targets were measured in a postabsorptive state). *(B)*, *(D)*, *(F)*, *(H)*, *(L)*, and *(L)* show protein expression in EX and CTL after bed rest in the postabsorptive (BR‐PA) or 4 h postprandial state after ingestion of 15 g of milk protein (BR‐PP), expressed as the fold change from levels measured in CTL after bed rest, which was normalized to a value of 1. For all panels, boxes represent the 25th to 75th percentiles, error bars represent SEM, and horizontal lines and crosses within boxes represent median and mean values, respectively (*n* = 9 for all targets except Akt, mTOR, rpS6 (*n* = 8)). Significance was set at *P* < 0.05. Following bed rest, there was a significant main effect of time on Akt^S473^ and mTOR^S2448^ expression, which was greater than CTL values after exercise prehabilitation (^#^ *P* < 0.05 for both, *(A)*A and *(C)*, respectively), with no difference between legs. Following bed rest, there was a significant main effect of time on 4E‐BP1^T37/46^ expression, which was significantly lower than CTL values after exercise prehabilitation (^#^ *P* < 0.05, *(E)*), with no difference between legs. Following exercise prehabilitation and bed rest, rpS6^S240/244^ expression was significantly higher in EX vs. CTL (*P < 0.05 for both, *(G)*). Following bed rest, rpS6^S240/244^ expression was significantly higher in EX vs. CTL in the postabsorptive and postprandial state (*P < 0.05 for both, *(H)*). Following bed rest, Akt^S473^ expression was significantly lower than the postabsorptive state in EX only (^#^ *P* < 0.05, *(B)*).

Following bed rest, there was a significant main effect of time on Akt^S473^ (*Figure* 6(A)) and mTOR^S2448^ expression (*Figure* 6(C)), which were greater than CTL values after prehabilitation (*P* < 0.01 and *P* < 0.001, respectively), with no difference between legs. Following bed rest, there was a significant main effect of time on 4E‐BP1^T37/46^ expression, which was lower than CTL values after prehabilitation (*P* = 0.04; *Figure* 6(E)), with no difference between legs. A significant main effect of leg was observed with rpS6^S240/244^ expression following exercise prehabilitation and bed rest (*Figure* 6(G), *P* = 0.004) and between the postabsorptive and postprandial states following bed rest (*Figure* 6(H), *P* = 0.033). Post‐hoc analysis revealed that, following exercise prehabilitation and bed rest, rpS6^S240/244^ expression (*Figure* 6(G)) was significantly higher in EX vs. CTL (*P* = 0.011 and 0.043, respectively). Further, following bed rest, rpS6^S240/244^ expression (*Figure* 6(H)) was significantly higher in EX vs. CTL in the postabsorptive and postprandial states (*P* = 0.043 and 0.013, respectively). Following bed rest, Akt^S473^ expression was significantly lower than the postabsorptive state in EX only (*P* = 0.015; *Figure* 6(B)). No significant group, time, or interaction effects were found for MuRF1 or MAFbx protein expression (*Figure* 6(I)–(L), all *P* > 0.05). Representative western blot images are presented in *Figure* S2.

## Discussion

Age‐related skeletal muscle loss (sarcopenia) is driven by impairments in muscle protein turnover that are accelerated during periods of disuse (i.e. hospitalization).^6^, ^9^ Muscle mass/attenuation is associated with length of stay in hospital, risk of readmission, recovery of function, and mortality.^31^ Given the treatment costs associated with sarcopenia,^32^ and the increasing prevalence of this condition,^3^ attenuating muscle mass loss in older individuals during an in‐hospital stay is crucial to maintain functional and metabolic health and lower the strain on health services (e.g. decreased bed occupancy and external care provision^33^). Compared with peri‐disuse or post‐disuse interventions, prehabilitation capitalizes on better patient health and could potentially benefit a number of clinically relevant outcomes.^17^ Here, we report that short‐term RET prehabilitation augmented iMyoPS over the week prior to 5 days of in‐hospital bed rest in older individuals. However, the relative decline in iMyoPS with bed rest was not offset by our prehabilitation protocol, and quadriceps muscle atrophy was not abated. In support of these findings, although postabsorptive and postprandial aMyoPS rates tended to be higher with RET prehabilitation, the net postprandial aMyoPS response was not enhanced/maintained.

The removal of muscle contractile activity in older individuals undergoing bed rest results in blunted rates of postprandial^8^ and postabsorptive aMyoPS,^34^ which drives the reduction in iMyoPS rates (i.e. incorporating postabsorptive and postprandial aMyoPS over a longer‐term) reported during disuse events.^29^, ^35^ RET is a potent stimulus for aMyoPS that, when undertaken routinely, augments iMyoPS for muscle protein accrual.^36^, ^37^ A single bout of RET enhances postprandial aMyoPS for at least 24 h afterwards,^38^ whilst the accumulation of six bouts of low‐load high‐volume RET over 14 days augments postprandial aMyoPS in older individuals, when measured 72 h after the final bout.^16^ Thus, the present study sought to determine whether four sessions of moderate‐load RET prehabilitation over 7 days (higher total volume and a shorter time frame than our earlier work^16^) would rescue iMyoPS, and consequently muscle mass, through repeatedly elevating postprandial aMyoPS in older individuals during bed rest. As expected, RET increased iMyoPS by 23% over the prehabilitation phase compared with the non‐exercised CTL, analogous to the increase in iMyoPS reported in younger adults with several bouts of RET.^25^, ^39^ Interestingly, the gene expression of p70S6K and mTOR increased above basal values during bed rest in CTL only, whilst mTOR gene expression during bed rest was greater in CTL vs. EX. These data suggest divergent mechanisms of regulation during bed rest between EX and CTL, reinforcing the observed difference in absolute iMyoPS between legs. Nevertheless, iMyoPS rates decreased significantly, and to the same relative extent, in both EX (−26%) and CTL (−22%) during bed rest. Furthermore, with the exception of rpS6, alterations in the protein phosphorylation of mTOR, Akt, and 4E‐BP1 with bed rest did not differ between EX and CTL. Collectively, the similar relative changes to iMyoPS and anabolic signalling expression over the bed rest period in CTL and EX might partially explain the similar degree of quadriceps muscle loss as measured by MRI. Interestingly, rpS6 phosphorylation was greater in EX vs. CTL after prehabilitation and bed rest, but this clearly did not impact on the extent of disuse atrophy.

The repeated daily stimulation of postprandial aMyoPS over time is considered the main locus of muscle mass regulation, which is further enhanced by RET. Prehabilitation could, therefore, provide a stimulus to bolster/retain muscle anabolic sensitivity to subsequent feeding occasions during bed rest. The ability of prehabilitation to chronically augment postprandial aMyoPS was investigated after the final day of bed rest. Ingestion of 15 g of milk protein isolate (~0.19 g/kg/BW) significantly increased aMyoPS in EX only, whilst no increase was observed in CTL. The latter aligns with observations of muscle anabolic resistance in older individuals after 5 days of bed rest without prior RET.^8^ On the other hand, although a trend for higher postabsorptive (~32%; *P* = 0.054) and postprandial (~31%; *P* = 0.052) aMyoPS rates was observed in EX vs. CTL, the relative increase in aMyoPS from the postabsorptive to postprandial state did not differ between legs. Furthermore, with the exception of Akt, there was no postprandial change in anabolic signalling phosphorylation in EX or CTL, and no differences in postprandial anabolic signalling between legs. Therefore, it appears that moderate‐load high‐volume RET prehabilitation, undertaken over 7 days, was insufficient to favourably influence postprandial aMyoPS stimulation or anabolic signalling over a subsequent 5 day bed‐rest period in older individuals. We acknowledge that any potential effect of RET prehabilitation on postprandial aMyoPS and anabolic signalling responsiveness may have dissipated over the course of bed rest and been absent by the time of assessment (6 days after the final bout). This opens the possibility that short‐term RET prehabilitation could alleviate muscle anabolic resistance and atrophy in older individuals during very short periods of disuse.^16^, ^40^

The possibility that net muscle protein deposition during RET prehabilitation could counteract muscle loss during subsequent disuse is based on the assumption that all synthesized proteins are incorporated into bound myofibrillar protein and that muscle protein breakdown (MPB) remains unchanged. If this holds true, the absolute increase in iMyoPS over 7 days of prehabilitation in the present study (2.8% or 0.4% daily) would have buffered against muscle mass loss during bed rest (~0.7, 3.3% and 2.4% at 40%, 60%, and 80% of muscle length, respectively). Unfortunately, we were unable to perform MRI scans at baseline to better understand the influence of short‐term RET prehabilitation on quadriceps CSA. A more viable explanation for the dissociation between elevated iMyoPS in EX and the observed muscle loss is the redirection of newly synthesized proteins toward RET‐induced muscle damage repair rather than muscle hypertrophy per se, as has been reported in younger men in response to a similar RET stimulus as that used herein.^41^ Furthermore, given that a single bout of high‐volume RET elevates MPB over 24 h post‐exercise,^42^, ^43^ a cumulative MPB response over the 7 day prehabilitation phase in the present study would detract from overall net muscle protein balance. Nonetheless, we did not observe any difference in the protein expression of the ubiquitin ligases, MAFbx or MuRF1, between EX and CTL after prehabilitation. Paradoxically, MAFbx gene expression was lower after prehabilitation in EX vs. CTL, suggestive of attenuated MPB. However, in light of evidence suggesting that MAFbx expression may increase in the immediate hours after RET, before falling below basal levels at 24 and 72 h post‐exercise,^44^ this finding may be more indicative of active remodelling processes in EX vs. CTL. Additional biopsy and blood sampling would have allowed us to better reconcile the role of muscle damage and proteolytic signalling in remodelling processes during short‐term exercise prehabilitation and bed rest. Notwithstanding, it is unlikely that our short‐term RET prehabilitation programme was sufficient for the level of net muscle protein accretion that would effectively cancel out the subsequent muscle loss during bed rest in older individuals.

Whilst impaired aMyoPS is regarded as the primary mechanistic driver of disuse‐induced muscle atrophy, evidence of a transient up‐regulation in genes associated with proteolysis in the first several days of disuse in older^8^, ^45^ implicates a role of elevated MPB in disuse atrophy. Given the effect of RET on MPB,^42^, ^43^ it is possible that short‐term RET prehabilitation may have influenced the MPB response to bed rest in older individuals in the present study. In contrast to Tanner *et al*.,^8^ we did not observe an increase in the gene or protein expression of MAFBX and MuRF1, after 5 days of bed rest in older individuals, despite comparable levels of muscle loss. However, we did observe an increase in myostatin, a negative regulator of muscle mass associated with MPB,^46^ in EX and CTL. Based on these findings, it is not possible to determine the time course of change in MPB over 5 days of bed rest in older individuals, or the contribution of MPB to the observed muscle loss. Regardless, there is no evidence to suggest that MPB differed between legs. Given that the relative change in iMyoPS and aMyoPS during bed rest was similar between EX and CTL, any difference in MPB between legs would have influenced overall net protein balance and muscle atrophy.

Mobility during hospitalization is a central modifiable factor in preventing in‐hospital functional decline and post‐discharge adverse outcome in older adults.^47^ Average daily step‐count in hospitalized older patients is ~600–1000 during a 5 to 6 day inpatient stay,^48^, ^49^ whilst >80% of time is typically spent lying and only ~3% standing or walking.^50^ In the present study, step‐count in older individuals during 5 days of bed rest was ~98% lower than habitual levels, and sedentary time increased at the expense of a reduction in activity levels. These very low step‐count and activity data were achieved by introducing a wheelchair to replace walking for most basic tasks and, hence, are more reflective of in‐hospital values for frail older individuals with functional limitations.^51^ The dramatic alterations in activity during bed rest resulted in a loss of quadriceps CSA at 40%, 60%, and 80% of muscle length (−0.7%, 3.5%, and 2.8%, respectively), which is broadly comparable with reports of an ~4% reduction in dual X‐ray absorptiometry‐derived whole‐leg lean mass following 5 days of bed rest in older individuals.^8^ Congruent with the present data, greater quadriceps atrophy at the proximal end of the muscle has been reported toward younger individuals during prolonged bed rest.^52^ A possible explanation for the heterogeneity in quadriceps and vastus lateralis atrophy at various muscle lengths may relate to differences in the function of certain muscle sub‐regions in daily life and their consequent change in activity during bed rest. However, the relationship between quadriceps atrophy and anatomical length may depend on age status and the model of disuse, as others have observed distal quadriceps atrophy at 20% of muscle length in younger individuals after 7 days of leg immobilization.^40^ The decrease in quadriceps CSA was not confirmed at the microscopic level, which may have been due to the relatively small sample size. Indeed, similar observations of a reduction in quadriceps CSA, but not fibre CSA, with short‐term bed rest have been reported.^53^ Another possible explanation could be that biopsy samples were obtained in a distal to proximal orientation (~2–3 cm between biopsies). As fibre CSA is not constant throughout the muscle length, it is imperative for future studies to sample skeletal muscle from the same location in order to track changes in fibre CSA with disuse.

Dietary protein intake stimulates aMyoPS and, therefore, plays a pivotal role in muscle mass maintenance in ageing and disuse. Higher protein intakes are associated with greater long‐term lean mass retention in older individuals,^54^ whereas a substantial increase in protein intake, on top of a diet providing the protein recommended dietary allowance or lower (≤0.8 g/kg/BW/day), attenuates muscle loss in older individuals during bed rest.^55^ To mimic a typical inpatient stay in the present study, we provided participants with a choice of typical hospital meals/snacks. Protein intake during bed rest averaged 1.0 g/kg/BW/day, which was ~23% lower than habitual levels but greater than a real‐world hospital setting, where older patients are often protein malnourished.^56^, ^57^ The reduction in total energy and dietary protein intake during bed rest in the present study may be reflective of reduced energy requirements and/or lower nutritional quality of hospital meals.^56^, ^58^ In support of the latter, dietary fibre intake was lower than habitual level during bed rest. Given that an increase in dietary protein during short‐term bed rest, on top of a diet containing >1.0 g/kg/BW/day, does not attenuate muscle disuse atrophy in healthy older men,^59^ we contend that the reduction in dietary protein intake from habitual levels during bed rest would not have accelerated muscle loss. Because the majority of food intake during bed rest came from pre‐prepared mixed‐ingredient meals, we were unable to determine potential changes in dietary protein quality. This point is noteworthy, as recent evidence suggests that improving dietary protein quality during short‐term bed rest offsets the decline in muscle mass and function in older individuals.^60^

Although daily energy intake (EI) during bed rest was lower than habitual levels, the dramatic reduction in physical activity (and energy expenditure) may have left participants in a hypercaloric state, which may explain the observed increase in body fat. Thus, we cannot discount the notion that a hypercaloric state and increased fat mass could have influenced muscle atrophy. However, recent work showed that high‐fat overfeeding exacerbated muscle amino acid balance but did not aggravate muscle atrophy during short‐term forearm immobilization in young individuals.^61^ Furthermore, despite the increase in body fat after bed rest in the current study, lipid profiles remained unchanged. This is important, as elevated circulating lipids may underlie the postprandial muscle anabolic resistance reported in obese older adults.^6^, ^62^ Thus, we speculate that the alterations in macronutrient/EI during bed rest had minimal impact on the observed muscle atrophy, although further clarification is required. The disconnect between body fat and lipid profile changes with bed rest may be explained by the limitations of BIA for body composition assessment. Pre‐bed‐rest and post‐bed‐rest BIA scans were conducted in the morning and afternoon, respectively (owing to logistical issues in the testing schedule), which could have led to slight discrepancies in fluid status and an overestimation in fat mass.^63^ Irrespective, any influence of alterations in macronutrient/EI during bed rest on muscle loss would have impacted EX and CTL similarly. Taken together, the alterations in macronutrient/EI observed during bed rest in our healthy older cohort reinforce the need to develop appropriate nutritional practices to support whole‐body and muscle health during an in‐hospital stay.

Although short‐term RET prehabilitation did not offset disuse‐induced muscle atrophy, there may be potential benefits for metabolic health. Bed rest and inactivity induce rapid insulin resistance and glucose dysregulation,^9^, ^53^, ^64^ which may underpin the progressive decline in metabolic health with advancing age.^65^ In contrast, plasma insulin, glucose, or HOMA‐IR did not significantly change from pre‐bed‐rest to post‐bed rest in the present study, although there was a trend for an increase in HOMA‐IR. Thus, it is possible that short‐term RET prehabilitation might offer some protection against disuse‐induced declines in peripheral insulin sensitivity and glucose regulation, a process that usually occurs after the first few days of inactivity.^66^ The potential for unilateral RET prehabilitation to support whole‐body insulin sensitivity in older adults during short‐term disuse has important clinical implications and requires further exploration.

In conclusion, the present study is the first to investigate the influence of short‐term RET prehabilitation on muscle morphology and associated regulatory mechanisms in older individuals during a period of disuse. Four bouts of single‐leg high‐volume RET, performed over 7 days prior to 5 days of bed rest, augmented iMyoPS as compared with the non‐exercised leg. However, the relative reduction in iMyoPS during bed rest was similar between legs, as was the loss of quadriceps muscle mass. RET prehabilitation had no clear influence on relative postprandial aMyoPS stimulation compared with the non‐exercised leg, when measured at the end of bed rest. The findings suggest that prior RET of sufficiently high‐volume and duration may be required to build a muscle mass reserve to buffer against the atrophic response to a typical inpatient hospital stay. Irrespective, the appreciable loss of muscle mass in older individuals undergoing bed rest, reinforces the need to implement strategies during disuse events to protect muscle mass, function, and whole‐body metabolic health.^67^ Whether short‐term RET prehabilitation can protect postprandial muscle anabolism and offset muscle atrophy in older individuals during a very short bed‐rest period (i.e. ≤3 days) remains to be seen, as do the potential benefits of RET prehabilitation on disuse‐induced insulin resistance.

## Conflict of interest

None of the authors have any conflicts of interest to disclose.

## Funding

This work was supported by an award from the Biotechnology and Biological Sciences Research Council to L.B. (BB/N018214/1) and the NIHR Clinical Research Facility in University Hospitals Birmingham NHS Foundation Trust, Birmingham. The authors of this manuscript certify that they comply with the ethical guidelines for authorship and publishing in the *Journal of Cachexia, Sarcopenia and Muscle*.^68^

## Supporting information


**Figure S1:** Representative immunofluorescence microscopy images of muscle fibre‐type cross‐sectional area. Images were obtained from the leg that underwent short‐term resistance exercise prehabilitation (EX) and the non‐exercised control leg (CTL), immediately prior‐to (pre; A and C) and following (post; B and D) 5‐days of bed rest in older individuals. Muscle sections were marked with MHCI (red stain), MHCII (green stain) and WGA (i.e. cell membrane; blue stain) with 20x magnification. Scale bars are 50 μm.
Figure S2: Representative images of signalling protein expression measured via western blot. Images were obtained at the end of 7‐days of prehabilitation (PREHAB) and at the end of 5‐days of bed‐rest in the postabsorptive (BR‐PA) or 4 h postprandial state after ingestion of 15 g of milk protein (BR‐PP). Samples were obtained from the leg that underwent short‐term resistance exercise prehabilitation (EX) and the non‐exercised control leg (CTL). The molecular weight (MW) of each target is detailed on the right of the images (kDa).

## Data Availability

The datasets generated during and/or analysed during the current study are not publicly available but are available from the corresponding author on reasonable request.
