# Supplementary figures and images for: The effect of short‐term exercise prehabilitation on skeletal muscle protein synthesis and atrophy during bed rest in older men
Source: J Cachexia Sarcopenia Muscle. 2020 Dec 21;12(1):52–69. doi: 10.1002/jcsm.12661 (PMC7890266; doi:10.1002/jcsm.12661)

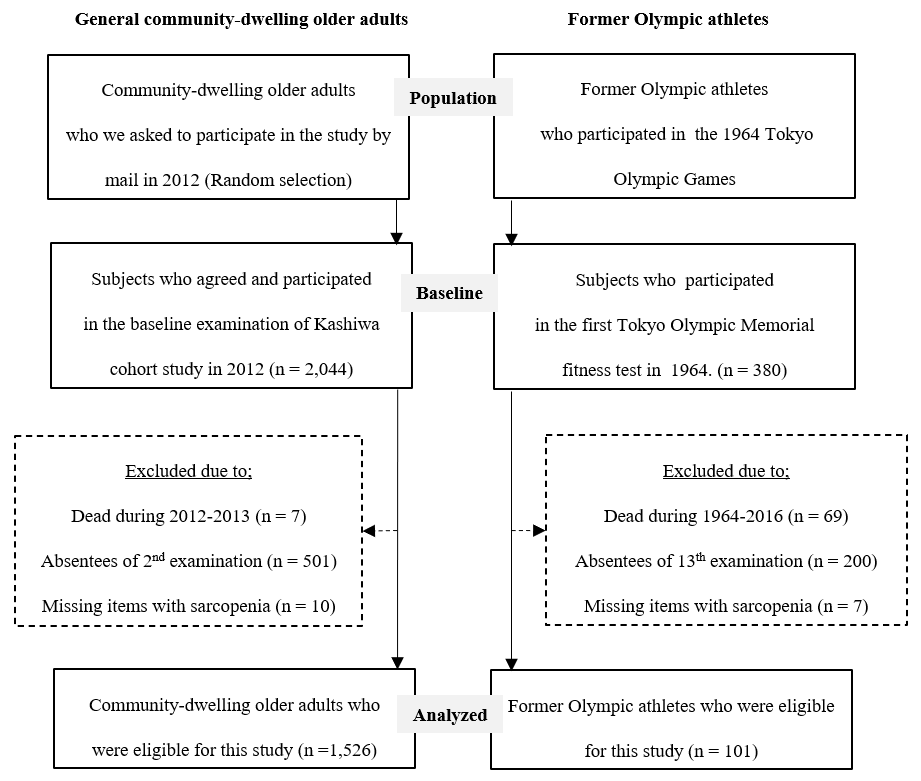

Supplement: Supplementary file 1 — Figure S1: Representative immunofluorescence microscopy images of muscle fibre‐type cross‐sectional area. Images were obtained from the leg that underwent short‐term resistance exercise prehabilitation (EX) and the non‐exercised control leg (CTL), immediately prior‐to (pre; A and C) and following (post; B and D) 5‐days of bed rest in older individuals. Muscle sections were marked with MHCI (red stain), MHCII (green stain) and WGA (i.e. cell membrane; blue stain) with 20x magnification. Scale bars are 50 μm. Figure S2: Representative images of signalling protein expression measured via western blot. Images were obtained at the end of 7‐days of prehabilitation (PREHAB) and at the end of 5‐days of bed‐rest in the postabsorptive (BR‐PA) or 4 h postprandial state after ingestion of 15 g of milk protein (BR‐PP). Samples were obtained from the leg that underwent short‐term resistance exercise prehabilitation (EX) and the non‐exercised control leg (CTL). The molecular weight (MW) of each target is detailed on the right of the images (kDa). [file JCSM-12-52-s001.docx]
